# Editorial: Women in gastrointestinal cancers, volume II: 2022

**DOI:** 10.3389/fonc.2023.1192814

**Published:** 2023-05-11

**Authors:** Nadia M. Hamdy, Lisardo Bosca, Sukh Mahendra Singh, Srinivasa Reddy Bonam, Igor Kiss, Divya P. Kumar, Aditi Banerjee

**Affiliations:** ^1^ Department of Biochemistry, Faculty of Pharmacy, Ain Shams University, Cairo, Egypt; ^2^ Alberto Sols Biomedical Research Institute, Faculty of Medicine, Autonomous University of Madrid, Madrid, Spain; ^3^ School of Biotechnology, Banaras Hindu University, Varanasi, India; ^4^ Department of Microbiology and Immunology, University of Texas Medical Branch, Galveston, TX, United States; ^5^ Department of Comprehensive Cancer Care, Masaryk Memorial Cancer Institute and Faculty of Medicine, Masaryk University, Brno, Czechia; ^6^ Department of Biochemistry, JSS Medical College, JSS Academy of Higher Education and Research, Mysore, India; ^7^ Department of Pediatrics, University of Maryland, School of Medicine, Baltimore, MD, United States

**Keywords:** women in science, personalized medicine, *in silico*/bioinformatics, CRC (colorectal cancer), gastrointestinal (GI) cancers

This editorial presents the inaugural Frontiers in Oncology (FIO) “*Women in Gastrointestinal Cancer, volume II: 2022*” series of article collections. The Research Topic collection highlights the diversity of research performed across the entire breadth of oncology research by women scientists pursuing STEM careers. Nine research articles, from the fourteen submitted to FIO within this section, have been published under this Research Topic by 92 authors with the aim of presenting advances in theory, experiment, and methodology and applications to compelling problems related to gastric cancers, such as gastrointestinal (GI) cancer, and hepato-pancreatic-biliary (HPB) cancers. One of the published articles is a brief research report article titled “the BET inhibitor JQ1 potentiates the anticlonogenic effect of radiation in pancreatic cancer cells”. Another is a case report article titled “*a female with synchronous multiple primary malignant tumors in the esophagogastric junction, duodenum, and pancreas: Case report and review of the literature*”. There is also a methodology clinical trial article titled “*implementing pharmacogenetic testing in gastrointestinal cancers (IMPACT-GI): Study Protocol for a Pragmatic Implementation Trial for Establishing DPYD and UGT1A1 screening to guide chemotherapy dosing*”. Four out of the nine published articles are original research articles exploring the tumor–immune interactions in the tumor microenvironment (TME) and identifying new prognostic and/or therapeutic biomarkers to assist in novel decoding mechanism(s) of tumor immunotherapy, as well as being confined with personalized treatment based on geno/phenotypic evaluations. These four articles are, first, “*Evaluation of Galanin Expression in Colorectal Cancer (CRC): An Immunohistochemical (IHC) and Transcriptomic Study*”; second, “*Swimming Impedes Intestinal Microbiota and Lipid Metabolites of Tumorigenesis in Colitis-Associated Cancer*”; third, “*The Novel LncRNA WASH5P Inhibits CRC Carcinogenesis via Targeting AKT Signaling Pathway*”; and fourth, “*Detection of Glycosylated Markers from Cancer Stem Cells With ColoSTEM Dx Kit for Earlier Prediction of Colon Cancer Aggressiveness*”. Moreover, there are two review articles titled “*Prospective Medicinal Plants and Their Phytochemicals Shielding Autoimmune and Cancer Patients Against the SARS-CoV-2 Pandemic: A Special Focus on Matcha*” and, a systematic review, “*Interactions of CRC, Dietary Fats, and Polymorphisms of Arachidonate Lipoxygenase and Cyclooxygenase Genes*”. The former review could be considered a study about the importance of herbal medicine (matcha) for protecting immunocompromised cancer patients against SARS-Cov-2. One experimental study used mice to test swimming as a physical activity that could influence metabolism and cancer susceptibility.

Most of the published articles performed bioinformatics and *in silico* analyses using several databases, either before the practical/experimental work or after for confirmation.

Cancer in general was mentioned in one case report paper, gastrointestinal cancers were addressed in three papers, and four papers presented CRC; however, pancreatic cancer was studied in only one article.

The emerging importance of *cancer-personalized treatment plans* is addressed in three research articles, in which the growing significance of cancer-tailored treatment strategies or personalized medicine is discussed.

It was claimed in a systematic review article by Gholamalizadeh et al. that dietary fat has an impact on the risk of CRC, which may be significantly influenced by the polymorphism of the genes arachidonate lipoxygenase (*ALOX*) and cyclooxygenase (*COX*). After further validation, it was suggested that dietary advice regarding fats for CRC prevention might be adopted based on the *ALOX* and *COX* personal genotypes.

Regarding the metabolism of fat and lipids, the role of physical activity was, interestingly, tested with the use of mice by Wang et al. by studying the protective effect of swimming against colitis-associated cancer (CAC). They claimed that swimming interferes with the connection between colonic lipid metabolites and prostaglandin E2 to its receptor signaling (PGE2/EP2 signaling). Swimming-induced genera given to the mice and probiotics increased the intestinal short-chain fatty acids (SCFAs) linked to the significant anti-inflammatory and anti-tumorigenic effects of swimming. Moreover, they were found to promote glycerophospholipid and choline metabolism in cancer cells. Therefore, the authors concluded that swimming is a potent preventive measure against CAC and implied that the differential lipid metabolites screened in the experiment are candidates for medicine with anti-inflammatory and tumorigenesis prevention properties. However, the results need further experimentation validation to identify/confirm their molecular basis.

Long non-coding RNAs (lncRNAs) and their differential expression levels have been implicated in the development and progression of CRC; thus, they are extensively studied nowadays. One article published by Wei et al. studied Wiskott–Aldrich Syndrome Homolog 5 Protein family (WASH5P). Compared to healthy controls, WASH5P expression was dramatically downregulated in CRC cell lines and tissues. The proliferation, invasion, and migration of CRC cells may be markedly reduced by the ectopic expression of WASH5P in these cells. When this happens, WASH5P overexpression can drastically reduce protein kinase B (AKT) activation by preventing AKT phosphorylation.

Talaat et al. performed an IHC and transcriptomic study of the neuropeptide human galanin to address its role in CRC. Galanin, which is widely distributed in the colon tissue and expressed in many cancers including CRC, was identified as a potential CRC-negative biomarker in this study. Galanin expression downregulation in the CRC was connected to cell cycle and cell division, autophagy, transcriptional regulation of TP53, immune system response, and advanced CRC staging.

Blondy et al. used the ColoSTEM Dx IHC kit developed by Carcidiag Biotechnologies to precisely detect glycosylated markers from cancer stem cells (CSCs) by identifying the glycan patterns that CRC stem cells (over)express. As a result, it provides a ground-breaking clinical tool for earlier tumor aggressiveness prediction, and it is of prognostic value for therapy response assessment in CRC patients.

Again, Varughese et al. reported the importance of pharmacogenetic (PGx) testing adoption in routine clinical care. Their current clinical trial (https://clinicaltrials.gov/ct2/show/NCT04736472) was conducted for “Implementing pharmacogenetic (PGx) testing in gastro-intestinal cancers IMPACT-GI Study protocol for a pragmatic implementation trial for establishing germline *DPYD* and *UGT1A1* variants”. They established that when tested/screened individually, these variants are associated with reduced enzyme activity, and when being tested/screened, they will guide 5-fluorouracil, capecitabine, and irinotecan chemotherapy dosing to patients at high risk of severe chemotherapy-induced toxicity. Furthermore, the dihydropyrimidine dehydrogenase gene *(DPYD)* and UDP-glucuronosyltransferase 1A1 *(UGT1A1)* polymorphisms are associated with lower enzyme activity—dihydropyrimidine dehydrogenase (DPD) for breaking down 5FU and the UGT1A1 enzyme for inactivating the active metabolite of irinotecan.

Garcia et al.’s study addressed the use of the BET inhibitor JQ1 in pancreatic cancer cells with ionizing radiation (IR) as a combined potentiating treatment option before surgery. JQ1 decreases the expression of the DNA repair protein RAD51 in cancer cells by potentiating the anti-clonogenic effect of IR and increases cancer cell DNA damage. Therefore, Garcia et al. recommended that patients with borderline respectable pancreatic cancer would benefit from using BETi JQ1 + IR as a treatment option pre-surgery

Du et al. suggested, for patients with multiple primary cancers (MPCs), a personalized treatment plan set by a committed multidisciplinary healthcare team with an assessment of all options at various disease/treatment stages along the disease trajectory. This suggestion was made based on their case report and literature review of a female with synchronous multiple primary esophagogastric junction adenocarcinoma, duodenal adenocarcinoma, and pancreatic ductal adenocarcinoma (PDAC), which had not been reported in the literature before.

Finally, one interesting review article by Kiriacos et al. addressed the impact of matcha as a prospective medicinal plant and novel potential protective and therapeutic phytochemical agent for cancer and immunocompromised patients during the SARS-CoV-2 pandemic. They claimed that matcha is a “tri-acting herbal tea having a potent antitumorigenic effect, immunomodulatory role, and proven anti-SARS-CoV-2 activity”. The review mentioned the current status of patients with cancer and their autoimmune system after the emergence of SARS-CoV-2 variants. Moreover, they addressed the effects of all the available medicinal and edible herbs given to those patients, such as black and green tea ingredients and the different constituents of matcha in comparison to previously mentioned herbs.

## Author contributions

NH and AB were the associate editors, and DK was the co-associate editor of the current Research Topic. NH wrote and revised the editorial paper text. LB, SS, SB, and IK acted as guest editors for one paper each in the FIO Research Topic: Women in GI tumors Vol II: 2022. All authors contributed to the article and approved the submitted version.

